# Acceptability of an Embodied Conversational Agent for Type 2 Diabetes Self-Management Education and Support via a Smartphone App: Mixed Methods Study

**DOI:** 10.2196/17038

**Published:** 2020-07-22

**Authors:** Shaira Baptista, Greg Wadley, Dominique Bird, Brian Oldenburg, Jane Speight, The My Diabetes Coach Research Group

**Affiliations:** 1 The University of Melbourne Melbourne Australia; 2 The Australian Centre for Behavioural Research in Diabetes Melbourne Australia; 3 The University of Queensland Brisbane Australia; 4 see Authors' Contributions

**Keywords:** embodied conversational agent, type 2 diabetes, mobile apps, mHealth, smartphone, self-management, mobile phone

## Abstract

**Background:**

Embodied conversational agents (ECAs) are increasingly used in health care apps; however, their acceptability in type 2 diabetes (T2D) self-management apps has not yet been investigated.

**Objective:**

This study aimed to evaluate the acceptability of the ECA (Laura) used to deliver diabetes self-management education and support in the My Diabetes Coach (MDC) app.

**Methods:**

A sequential mixed methods design was applied. Adults with T2D allocated to the intervention arm of the MDC trial used the MDC app over a period of 12 months. At 6 months, they completed questions assessing their interaction with, and attitudes toward, the ECA. In-depth qualitative interviews were conducted with a subsample of the participants from the intervention arm to explore their experiences of using the ECA. The interview questions included the participants’ perceptions of Laura, including their initial impression of her (and how this changed over time), her personality, and human character. The quantitative and qualitative data were interpreted using integrated synthesis.

**Results:**

Of the 93 intervention participants, 44 (47%) were women; the mean (SD) age of the participants was 55 (SD 10) years and the baseline glycated hemoglobin A1c level was 7.3% (SD 1.5%). Overall, 66 of the 93 participants (71%) provided survey responses. Of these, most described Laura as being helpful (57/66, 86%), friendly (57/66, 86%), competent (56/66, 85%), trustworthy (48/66, 73%), and likable (40/66, 61%). Some described Laura as not real (18/66, 27%), boring (26/66, 39%), and annoying (20/66, 30%). Participants reported that interacting with Laura made them feel more motivated (29/66, 44%), comfortable (24/66, 36%), confident (14/66, 21%), happy (11/66, 17%), and hopeful (8/66, 12%). Furthermore, 20% (13/66) of the participants were frustrated by their interaction with Laura, and 17% (11/66) of the participants reported that interacting with Laura made them feel guilty. A total of 4 themes emerged from the qualitative data (N=19): (1) perceived role: a friendly coach rather than a health professional; (2) perceived support: emotional and motivational support; (3) embodiment preference acceptability of a human-like character; and (4) room for improvement: need for greater congruence between Laura’s words and actions.

**Conclusions:**

These findings suggest that an ECA is an acceptable means to deliver T2D self-management education and support. A human-like character providing ongoing, friendly, nonjudgmental, emotional, and motivational support is well received. Nevertheless, the ECA can be improved by increasing congruence between its verbal and nonverbal communication and accommodating user preferences.

**Trial Registration:**

Australian New Zealand Clinical Trials Registry CTRN12614001229662; https://tinyurl.com/yxshn6pd

## Introduction

Diabetes will affect 693 million people worldwide by 2045, most of whom will have type 2 diabetes (T2D) [1,2]. People with T2D can prevent or delay the onset and progression of diabetes-related complications such as heart attack, stroke, kidney failure, vision loss, and nerve damage through intensive management of blood glucose levels [3]. However, effective self-management is complex and difficult to implement and sustain in daily life. Consequently, many people with T2D are not able to achieve their recommended self-management targets [4].

For several decades, diabetes self-management education and support have been provided in person (one-to-one and group-based), with many trials and real-world studies demonstrating improved diabetes outcomes [5]. However, the high cost and resource requirements limit the reach and scalability of in-person programs [6]. Furthermore, ongoing in-person support for sustaining the recommended diabetes care targets is not feasible for most health care systems [4].

Considerable advances in technology related to smartphone apps (including voice recognition, natural language processing, and artificial intelligence capabilities) have led to an increase in the feasibility of using embodied conversational agents (ECAs) to provide education and support for the self-management of chronic conditions, including T2D [7]. An ECA is an animated conversational human-like character that simulates person-to-person conversation with appropriate dialog and human-like physical properties, including facial expressions and body movements [8-10]. ECAs are increasingly being used in a wide range of apps, providing support for mental health, web-based information seeking, medication taking, behavior change, and prevention of suicide [7,10-13].

Research on the acceptability of ECAs in self-management of chronic conditions is still in its infancy, with a small number of studies reporting high levels of acceptability of ECA-based interventions [13-15]. Trust, empathy, and expertise have been cited as essential components of diabetes education and support [16]. Similar expectations may exist when the intervention is delivered by an ECA. ECAs use facial expressions, body movements, and speech and can offer a natural and accessible means of communication. These characteristics of ECAs potentially improve engagement compared with a static character image, a nonrelational agent, or a text-only display [9,17,18]. ECAs may be perceived to provide additional motivational and emotional support, which has previously been described by people with diabetes as being as important to them as practical support [19]. Preliminary evidence suggests that ECAs are perceived to be less judgmental, less intimidating, and more likable than a human counterpart, resulting in participants feeling less guilty and more motivated by the interaction [13,14,17,18]. Collectively, this evidence suggests that ECAs may be effective in providing support for chronic disease management as they help to engage users by building a social and emotional relationship over time [9,18,20].

Some of the characteristics of ECAs that may affect their acceptability include users being deterred by a monotonous voice and repetitive messages [13,18,20]. Although ECAs are more engaging if they have human-like characteristics and engage in social dialog, this effect is mitigated if there is an unnatural dissonance between a character’s speech and the expected facial expressions and body movements of the ECA [17,21-23]. This phenomenon, coined the *uncanny valley* by Masahiro Mori in 1970, was supported by research suggesting that people have unpleasant impressions of artificial characters, such as ECAs that have an almost, but not perfectly, realistic human appearance [24,25]. Previous studies have also emphasized that the visual characteristics of an ECA are important as these affect the perceptions of trustworthiness and credibility, which can affect acceptability. For example, a more playful, cartoon-like character is perceived as being more friendly, whereas a more serious human-like character, dressed professionally, is usually perceived to be more appropriate for serious apps, such as self-management of chronic conditions [26].

Research on the acceptability of ECAs to deliver self-management support for chronic conditions via self-management apps has been limited primarily to short-term feasibility or pilot studies and to interventions that address only a single behavior. Other studies use static images rather than animations or have been conducted using desktop or laptop computers in laboratory settings rather than with personal smartphones used in everyday settings or *in the wild* [27,28]. Thus, this study aimed to address these gaps by investigating the acceptability of an ECA delivering self-management education and support to people with T2D in their everyday lives.

## Methods

### Study Design

A convergent study design was used where quantitative and qualitative data were collected at similar time points [29]. This study was conducted within the context of a randomized controlled trial to test the effectiveness of a T2D self-management smartphone app, My Diabetes Coach (MDC) [30,31]. The MDC study was conducted from 2014 to 2018 (Australia New Zealand Clinical Trials Registry ID ACTRN12614001229662). The study was approved by the University of Melbourne’s Human Research Ethics Committee (ethics ID 1442433).

### My Diabetes Coach

**Figure 1:**
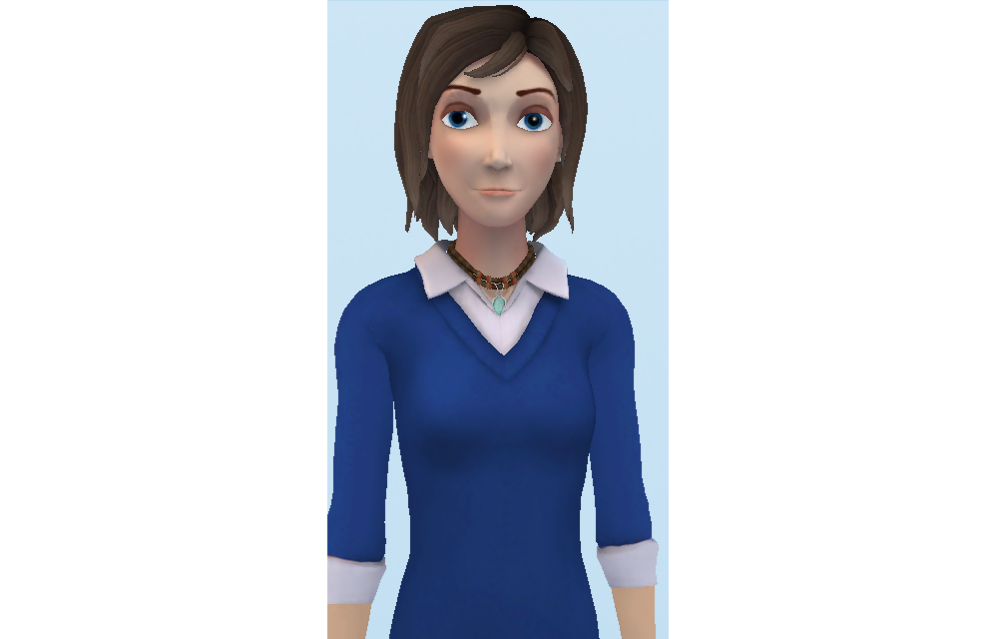
Laura, the embodied conversational agent.

MDC used an ECA called Laura (Figure 1) to deliver self-management education and support to adults with T2D. When users logged in for the first time, they were prompted to set up a regular time to complete weekly interactive sessions with Laura. During these conversations, Laura provided education, feedback and motivational support for blood glucose level monitoring, taking medication, physical activity, healthy eating, and foot care. The conversations were personalized to the individual’s self-management targets, physical fitness, and foot health using recommendations provided by his or her general practitioner.

The MDC app used voice recognition, prescripted conversational elements, and a sophisticated script logic enabling the user to interact with Laura in several predetermined variations, mimicking natural conversations. Laura’s voice and conversation were produced by a proprietary dialog engine (by Clevertar). Nonverbal behaviors were either explicitly scripted for each dialog, or, if no behavior was specified, they were selected randomly from a finite set of animations based on whether the character was speaking and the dialog duration. User responses from previous sessions dictated the direction of future sessions, enabling a high degree of personalization. The ECA’s appearance, conversational elements, back story, and accent were refined through several rounds of expert and user testing. Users were able to respond to Laura by touching an option on the screen or by speaking out one of the options on the screen when prompted to do so. Users also had access to a web-based discussion board and website (with additional diabetes resources) that could be accessed via the app as well as technical support from the research team. An excerpt from a conversation with Laura can be found on YouTube [32].

### Participants

Recruitment methods for the MDC trial are reported in the main outcomes paper (under review). Briefly, participants were recruited to the MDC trial from the general population in Australia via several recruitment strategies. Adults with T2D registered on the National Diabetes Services Scheme (NDSS) database; willing to be contacted about research; and living in New South Wales, Queensland, Victoria, and Western Australia were invited to participate by the NDSS via mail and email. The invitation letters were supplemented with media releases and targeted advertising on social media by several organizations (Diabetes New South Wales; Diabetes Queensland; Diabetes Victoria; Diabetes Western Australia; Bupa Australia, a health insurance provider; and the Australian Diabetes Educators Association).

For this study, participants from the intervention arm of the MDC trial, who had access to the MDC app, completed a survey at 6 months postbaseline, assessing several clinical and behavioral outcomes, including their interaction with the ECA, and a purposive subsample participated in subsequent interviews. All participants received a plain language statement describing the study and provided written consent.

### Data Collection

#### Demographic and Clinical Characteristics

The demographics and duration of diabetes (self-reported) were collected using web-based surveys at baseline. Glycated hemoglobin A_1c_ (HbA_1c_) is a pathology test assessing average blood glucose levels over the past 2 to 3 months, providing an indication of risk for long-term complications [33]. It was obtained, with participants’ consent, from their general practitioner.

#### Acceptability: Quantitative Data

At the 6-month follow-up, the participants completed a web-based survey that included 2 questions assessing the acceptability of the ECA. The first question assessed the perceptions of the ECA: “How well do the words below describe Laura?” The respondents rated a range of positive and negative traits (helpful, boring, friendly, competent, annoying, likable, trustworthy, and real) using a 5-point Likert-type scale ranging from *describes very well* to *describes very poorly*. The second question asked, “How did interacting with Laura make you feel?” The respondents selected from a list of descriptive emotions (happy, confident, hopeful, motivated, worried, guilty, frustrated, and comfortable) and were asked to select all that applied. For both questions, positive and negative words were randomly sequenced to minimize response bias. The descriptive adjectives were chosen based on the literature on evaluating ECAs and on working alliances between ECAs and users [34].

#### Acceptability: Qualitative Data

In-depth, semistructured qualitative interviews were conducted from October 2017 to February 2018. Most participants had, at that stage, completed the 6-month survey but were still actively using the app. Purposive sampling of survey respondents was used to identify interviewees who varied in terms of the duration of diabetes, gender, age, and baseline familiarity with apps.

The interview guide was developed by the first author (SB) and used exploratory questions and probes, with feedback from other members of the research team (BO, GW, and JS) based on the research question and findings from the current literature [8,9,14,18,21-23,26,27,35-38]. The guide explored a variety of topics, including experience at diagnosis; self-care behavior before using the MDC app; users’ experiences with the MDC app, including when, where, and how it was used; changes to self-management practices as a result of using the MDC app; initial impression of the ECA Laura and changes over time; perceptions of her role in self-management, and her perceived personality characteristics. The data relating to the acceptability of Laura are presented here, with the other findings published separately.

The interviews were conducted by telephone (by SB) and recorded using a cloud architecture solution from the CTI Group using their SmartInteraction Suite of recording software. During each interview, SB used exploratory questions and probes (from the interview guide) and noted points of interest, using these as further probes. Immediately after each interview, SB prepared a written summary of the interview and any relevant observations. These were used to communicate interim findings to the research team. When appropriate, additional questions were added to the interview guide, enabling further exploration of the issues raised by participants that were relevant to the research aims. These notes were also used to aid in the meaningful interpretation of data during data analysis.

### Data Analysis

The quantitative data were analyzed using IBM’s SPSS Statistics 25 package. Descriptive statistics were computed for demographic and clinical characteristics and 2 questions assessing the ECA. The qualitative data were transcribed, deidentified, and thematically analyzed using NVivo 11, following the first 5 steps of Braun and Clarke’s methodology [39,40]. The integration of the quantitative and qualitative data was achieved at the interpretation stage by comparing the findings from the surveys and the semistructured interviews. In practice, this involved referring to and using the qualitative data to help interpret, triangulate, and add meaning to the quantitative data. This process was iterative, with input from several researchers (SB, GW, BO, and JS). This integration of quantitative and qualitative data enabled further validation of the findings and increased their explanatory value [41]. The narrative of the results is blended with embedded quotes from several sources to make the results more readable while using as much evidence as possible. An anonymized coding system—participant identity number (IDX): sex (male, M; female, F): age (years)—was used to identify the source of each quote (in parentheses after each quote).

## Results

### Sample Characteristics

Of the 93 MDC trial participants in the intervention arm, 66 (71%) participants provided responses at 6 months postbaseline, and 19 of these participated in the interviews. Table 1 details the characteristics of the 3 samples. Overall, 50% (33/66) of the survey respondents were women, and the mean age of the participants was 57 (SD 9) years and the mean baseline HbA_1c_ level was 7.1% (SD 1.4%) [33].

Those who completed the survey were significantly older (*P*=.03) and completed more interactions with Laura (*P*=.001) than those who did not complete the survey. No significant differences were observed between the interviewees and other participants in the intervention arm, except that the interviewees completed significantly more interactions with Laura (*P*=.001).

The mean duration of the interviews was 51 min (range 29-79 min).

Overall, participants found Laura to be acceptable and were positive in their appraisal of her and their interactions with her. Most respondents agreed that Laura was helpful (57/66, 86%), friendly (57/66, 86%), competent (56/66, 85%), trustworthy (48/66, 73%), and likable (40/66, 61%). Some participants described her as boring (26/66, 39%) and annoying (20/66, 30%; Multimedia Appendix 1). Participants were undecided about whether or not they thought Laura was realistic. Of the 66 participants, 26 (39%) participants agreed that Laura was real, 22 (33%) were undecided, and 18 (27%) disagreed. The participants’ responses to their interactions with Laura were positive overall, with many reporting that she made them feel motivated (29/66, 44%), comfortable (24/66, 36%), confident (14/66, 21%), happy (11/66, 17%), and hopeful (8/66, 12%). Notably, 20% (13/66) were frustrated by their interaction with Laura, and 17% (11/66) of the participants reported that interacting with Laura made them feel guilty. One participant reported feeling worried (Multimedia Appendix 2).

Overall, 4 themes were identified from the qualitative data: (1) perceived role—a friendly coach rather than a health professional; (2) perceived support—emotional and motivational support; (3) embodiment preference—acceptability of a human-like character; and (4) room for improvement—need for greater congruence between Laura’s words and actions. Table 2 provides an integrative synthesis of the findings, summarizing the 4 main themes emerging from the qualitative data, quantitative endorsement of the adjectives describing Laura and how the interaction made the participants feel, and exemplars of the qualitative data. The 4 themes are described in detail below.

**Table 1:** Demographic and clinical characteristics of the total sample and interviewed sample.

Participant Characteristics		My Diabetes Coach trial population (intervention arm; n=93)		Six-month follow-up sample (n=66)		Interviewed participants (n=19)	
Gender (female), n (%)		44 (47)		33 (50)		8 (42)	
Age (years), mean (SD)		55 (10)		57 (9)		60 (8)	
**Education (highest level), n (%)**							
	Year 10		10 (11)		9 (14)		5 (26)
	Year 12 or apprentice		42 (45)		31 (47)		2 (11)
	Graduate or post graduate		41 (44)		26 (39)		12 (63)
**Employment status, n (%)**							
	Paid employment		59 (63)		41 (62)		7 (37)
	Retired		22 (24)		18 (27)		11 (58)
	Unemployed or other		12 (13)		7 (11)		1 (5)
**Duration of diabetes (years), n (%)**							
	<5		43 (46)		25 (38)		8 (42)
	5-10		29 (31)		23 (35)		8 (42)
	10-20		7 (8)		4 (6)		3 (16)
	Unknown		14 (15)		14 (21)		0 (0)
Baseline glycated hemoglobin A_1c_ (%), mean (SD)		7.3 (1.5)		7.1 (1.4)		6.8 (0.9)	
Baseline glycated hemoglobin A_1c_ (mmol/mol), mean (SD)		56 (44)		53 (30)		51 (20)	
**General app usage^a^ (reported at baseline), n (%)**							
	Multiple times per day		69 (74)		50 (76)		14 (74)
	Once a day		23 (25)		13 (20)		4 (21)
	Less than once a day		1 (1)		3 (5)		1 (5)
**Total interactions with Laura, mean (SD)**		18 (15)		23 (16)		36 (17)	

^a^General app usage at baseline represents the use of any app before participating in the My Diabetes Coach trial.

**Table 2:** Integrated results matrix.

Themes	Quantitative data: endorsement of adjectives	Qualitative data: exemplar quotes
Perceived role: Laura is more acceptable as a friendly coach than as a health professional	Laura was likable, n=40 (61%), friendly, n=57 (86%), and helpful, n=57 (86%) Interacting with Laura made me feel comfortable, n=24 (36%) Interacting with Laura made me feel guilty, n=11 (17%), and worried, n=1 (1%)	“A ‘neutral approach’ was ‘better’ because it ‘didn’t try and lean on any perceptions of authority.’” [ID04: M^a^: 44 years] “I was worried about making sure that I was within [my limits] knowing that I had to report to Laura!” [ID11: F^b^: 62 year]
Perceived support: Laura provides emotional and motivational support	Laura was trustworthy, n=48 (73%). Interacting with Laura made me feel confident, n=14 (21%), hopeful, n=8 (12%), and happy, n=11 (17%) Laura was competent, n= 56 (85%). Interacting with Laura made me feel motivated, n=29(44%)	“I needed somebody just to be there.” (ID15: F: 66 years) “(She) used to make me laugh...and that’s hard to do.” [ID18: M: 65 years] “She was keeping you on track and keeping you doing what you’re supposed to be doing.” [ID16: F: 57 years]
Character preference: Laura is engaging and her human-like character is appropriate	Laura was helpful, n=57 (86%) Laura was competent, n=56 (85%), and trustworthy, n=48 (73%). Laura made me feel confident, n=14 (21%), and comfortable, n=24 (36%)	“Instead of reading it, you’re hearing it and can read at the same time. Instead of just hearing some voice, you’re actually seeing [Laura] talk.” [ID05: M: 55 years] “I’m not sure I would have given the same level of credibility to, for example, a dog or a cat or something like that.” [ID04: M: 44 years]
Room for improvement: dissonance between Laura’s words and actions	Laura was annoying, n=20 (30%), boring, n=26 (39%), and not real, n=18 (27%) Interacting with Laura made me feel frustrated, n=13 (20%)	“She said something, but her hand gestures were exactly the opposite of what they should have been. Like, rather than a big gesture, where a big gesture is needed, there was a little gesture.” [ID08: F: 42 years]

^a^M: male.

^b^F: female.

### Theme 1: Perceived Role—Laura Is More Acceptable as a Friendly Coach Than as a Health Professional

When prompted about what role Laura was perceived to play in self-management support, some participants described Laura as “a ‘friendly’ coach” (ID11: F: 62 years) who was just “reminding me” of various diabetes self-management tasks. Furthermore, when asked about their perceptions of Laura, some participants described her with adjectives suggesting that she had a personality, such as “sassy” (ID15: F: 66 years), “friendly” (ID16: F: 57 years), “kind” (ID05: M: 55 years), and “intriguing” (ID06: M: 71 years). These findings may explain why most survey respondents described Laura as likable, friendly, and helpful and reported that interacting with Laura made them feel comfortable.

Conversely, other participants commented on how Laura reminded them of their health professional: “There were times when I would go and see my doctor, and I’d see Laura sitting there, because her gestures, her voices, and mannerisms are almost identical” (ID08: F: 42 years). Some participants “did not necessarily want to see an authority figure” (ID1: M: 63 years), saying, for example, that “I don’t need to be called into a doctor” (ID09: M: 71 years). Those who described Laura in similar terms to their health professionals did not warm up to Laura as they found her to be “patronizing,” “censorious,” and “authoritarian” (ID11: F: 62 years). For example, some described receiving her feedback as “having a mother-in-law in your pocket” (ID08: F: 42 years) and “feeling as though you’re getting a slap on the wrist” (ID02: F: 66 years) similar to “a recalcitrant child” (ID15: F: 66 years). Other negative descriptions of Laura were that she was “really young,” “super-skinny” (ID11: F: 62 years), and that she “talked at” people (ID13: M: 58 years).

Laura’s perceived role influenced the participants’ reactions to the support she offered. For example, participants who described Laura as being similar to a health professional reacted to this by “resisting” and “rebelling” against the “kind of authority” (ID11: F: 62 years) that Laura represented to them. One participant described how “feeling guilty” led him to “stop using” the app for a while (ID13: M: 58 years). Another participant commented on how she worried about negative feedback: “I was worried about making sure that I was within [my limits] knowing that I had to report to Laura!” (ID11: F: 62 years) Finally, one participant contemplated selecting her best readings to report to Laura to avoid “getting told off,” saying:

Do I record this one? It might be a bit high and she’s going to get upset with me.

[ID15: F: 66 years]

Conversely, those who perceived Laura to be less of an authority figure and more like a friendly coach as she “didn’t try and lean on any perceptions of authority, like for example, having a doctor in a white coat” were also more receptive to the support she offered. This is because they perceived her as having a more “neutral approach,” which was “better” because “a conversation between peers is more likely to be engaged with than one that references levels of authority” (ID04: M: 44 years).

The varied reactions of the participants to Laura may be linked to the inconsistencies between how Laura looked and how they expected her to act*.* For example, one participant said:

It's set up with this young, groovy woman who's going to help me, but she sounded like my GP who was telling me what to do. So, it's a kind of disconnect between how [Laura] looks and what she's actually saying.

ID13: M: 58 years

Finally, some participants described Laura’s role as an artificial entity as a positive trait, making them more receptive to receiving support from her. This is because they experienced judgment and blame for their condition from “real” people:

From the minute they meet you, just by the look of you, by the look of your appearance, they will judge you. That's one thing I don't like about real people because it happened to me.

ID03: F: 62 years

### Theme 2: Perceived Support—Laura Provides Emotional and Motivational Support

For many, Laura provided emotional support that the participants did not otherwise have:

I needed somebody just to be there. I see the hospital doctors every six months, I only see my local doctor when I need scripts or something. Apart from that, who do you talk to?

ID15: F: 66 years

Supporting this premise is the fact that many survey respondents thought that Laura was trustworthy, and interacting with her made them feel confident. Another example of how Laura provided emotional support is described by one participant who expressed how her humor helped him feel better:

[She] used to make me laugh when she used to stand there with her hands on her hips waiting for me sometimes. Like my wife is saying it was probably good because if you felt down or something it made you feel better. Well it definitely bought a smile to may face a lot of times and my wife said that’s hard to do.

ID18: M: 65 years

A small number of survey respondents reported that interacting with Laura made them feel happy, demonstrating some support for the premise that she may have helped alleviate some of the burden of care.

Laura also provided additional motivation through enhanced monitoring and positive reinforcement:

She was keeping you on track and keeping you doing what you're supposed to be doing and keeping you doing the check-ups and that sort of stuff.

ID16: F: 57 years

When I was doing the exercise section, she would ask for me to record how much exercise I was doing for the week and when I’d come back [and do it], I actually almost got a pat on the back from her. I wasn't trying to be impressive for (Laura), but I think it just gave you that little bit more incentive.

ID01: M: 63 years

Similarly, many survey respondents reported feeling more motivated after their interactions with Laura.

### Theme 3: Character Preference—Laura Is Engaging and Her Human-Like Character Is Appropriate

Interacting with Laura provided an additional dimension to the relational aspect of communication, resulting in reports of improved engagement:

instead of reading it, you're hearing it and can read at the same time. Instead of just hearing some voice, you're actually seeing [Laura] talk.

ID05: M: 55 years

Participants appreciated this additional dimension of communication, describing it as an attempt to “try and engage with you” and compared it with other apps where “you’re inputting information and you might get a summary,” but there was no “attempt to interact back with the user” (ID10: M: 49 years):

Laura was more personal so that's why I think I went on for the six months. The other apps were like just an impersonal graph or something, or just boxes where you put the things in.

ID07: F: 67 years

Some participants expressed a strong preference for Laura’s human-like character. Diabetes was described as “a human problem that should have a bit of stance and a bit of professionalism” (ID06: M: 71 years). Others thought that a nonhuman character such as a “fuzzy duck” or “Dobby the diabetes elf” (ID11: F: 62 years) would be better as it would be more “fun.” For these people, having “a character, even a fictitious character” was more “user friendly” and better than having “nothing there” (ID07: F: 67 years).

Participants who preferred a human-like character did not think a cartoon character could be taken seriously: “I’m not sure I would have given the same level of credibility to, for example, a dog or a cat or something like that” (ID04: M: 44 years). Two users put it as follows:

A cute puppy telling you that you got to exercise more or, you know, eat more greens, is going to be less convincing than a human. It just becomes a toy. Stick with somebody that looks like they know what they’re talking about. [Laura] fitted that bill.

ID17: M: 66 years

[A nonhuman character] would just make me want to throw the phone away completely! Because it's about a human interaction with someone who has information and resources about diabetes.

ID13: M: 58 years

However, there were those who did not care about what kind of character Laura was because she was “an inanimate object, not a person” (ID19: M: 59 years). Some did not “identify with or warm to Laura.” One participant said, “Laura had various statements [that were motivational] but I don’t have a relationship with Laura that caused me to value her opinion” (ID04: M: 44 years). Another participant said that although she “learnt from” Laura:

it’s not like if you went to your GP and you got your bloods done and it was physically down from the last six months, that’s a tangible quantity, but when it’s coming from an avatar, it didn’t really mean anything much.

ID12: F: 61 years

Some participants described being irritated by Laura’s “life” story, for example, when she said “I find that my family does such-and-such,” because she was pretending to be something she was not: “Don’t try and put it over me that this is a real person that I’m talking to” (ID16: F: 57 years). However, others liked Laura’s backstories:

Yeah, even though it's not real, but the way she talks about her kids and things like that. [I liked that] because it is more human.

ID06: M: 71 years

### Theme 4: Room for Improvement—A Dissonance Between Laura’s Words and Actions

When prompted about Laura’s appearance, speech, and mannerisms, the interviewed participants described Laura as being “just another robot” (ID09: M: 71 years) that they “could not connect to” as she was “not human enough.” Interacting with Laura, for many, depended on “how far along are you going to pretend.” As one participant put it, “I couldn't suspend belief that Laura wasn't this algorithm working out what she needed to say to me or not say to me” (ID13: M: 58 years).

The primary reason given for this perception was Laura’s “monotone” (ID08: F: 42 years) voice that sounded similar to a “mechanised reading mechanism” with “a strange cadence and inflection to some of her sentences” (ID11: F: 62 years). Another reason was her “artificial movements” (ID14: M: 66 years) and dissonance between what was being said and her body movements. According to one user:

She said something, but her hand gestures were exactly the opposite of what they should have been. Like, rather than a big gesture, where a big gesture is needed, there was a little gesture.

ID08: F: 42 years

This may have been the reason why some survey respondents reported feeling frustrated after interacting with Laura and why a reasonable proportion of participants described Laura as boring, annoying and not real, or were undecided about these descriptions.

Although it seems as though Laura was not an entirely successful ECA, participants were willing to overlook her shortcomings as they understood the intention behind Laura and appreciated the effort made to make her engaging: they were willing to “cut them some slack” because “at least it’s trying to be personable.” Moreover, “They’re trying to make her look [real]—I can understand what they’re trying to do” (ID17: M: 66 years).

## Discussion

### Principal Findings

Overall, the results suggest that an ECA is acceptable to people with T2D for the delivery of long-term self-management education and support. We found that people with T2D were willing to make compromises and adjust their expectations, while appreciating the effort of trying to create something more appealing and engaging than graphs and numbers on a screen. This implies that the increased interaction offered by an ECA may be valuable to users and a worthy avenue for developers to pursue when designing apps for people with chronic conditions such as diabetes [20].

Our findings corroborate earlier research suggesting that some users perceive an ECA to be less judgmental and more likable than a human counterpart [13,14]. This is an important finding as people with T2D often experience diabetes-related stigma, the consequences of which can include disengagement with or suboptimal self-care and diabetes-related health outcomes [42]. Suitably designed ECA support may be especially important in making people who experience diabetes-related stigma feel less judged and more open to sharing difficulties with self-management, thereby potentially increasing their engagement with appropriate self-care [43]. We also suggest that using supportive, nonblaming language is critical when designing ECAs for stigmatized conditions such as diabetes [44].

The results suggest several mechanisms through which an ECA may help establish and maintain a relationship with the user over time, such as increasing relational communication, providing ongoing emotional support and motivation, and alleviating some of the burden associated with chronic disease management through humor [18]. Our results suggest that another way to improve acceptability is to achieve a better match between an ECA’s appearance and users’ expectations of the ECA’s perceived role. For example, some participants expressed that diabetes is a serious human issue and viewed human-like characteristics as being more credible. Others expressed the desire to alleviate the burden of management by incorporating a fun character, supporting previous findings of a similar nature [26]. These varying opinions may reflect the nascent nature of the field and the fact that ECAs are not yet common.

Another related finding was that participants who perceived Laura to be a friendly coach were more open to receiving support from her when compared with those who perceived Laura to be similar to a health professional. The implication is that an ECA with a relaxed, friendly approach may be more successful in building a supportive relationship than an ECA that adopts a more authoritative role. Future attempts to develop ECAs for diabetes management could accommodate both viewpoints by striking a balance with a human-like, friendly, approachable character and avoiding patronizing messaging and mannerisms. More research is necessary to determine how expectations of users on the role that an ECA plays in self-management varies and how this informs their preference for the ECA’s character.

Another important consideration is just how *human* an ECA should be. Although participants reported a clear preference for a human-like character, which is supported by previous research [15], her presentation of a backstory might be a step too far as it did not seem credible to some participants, possibly because of an *uncanny valley* phenomenon [25]. This finding is supported by previous research on other relational agents whose personality traits and life stories are enjoyed by some users, whereas, to others, the attempt at making them too human-like is not appealing [18,45]. It will be interesting to explore attitudinal changes toward ECAs with personality as they become more common, and familiarity increases.

Our findings add to the mounting evidence that suggests that perfecting natural communication via congruence between verbal and nonverbal communication is critical to improving acceptability [20]. Nonverbal cues such as facial expressions, gaze, gestures, postures, and body movements have a deep impact on the process and outcome of communication, with approximately 65% of social meaning derived from nonverbal behavior [46]. Laura’s mannerisms and body movements were the main basis on which she had particular personality traits attributed to her, ranging from patronizing and censorious to funny and sassy. Although the MDC app attempted to create an ECA with natural communication, this effort was impeded by difficulties using the speech recognition function; lack of inflection in Laura’s voice; and a limited number of random body movements, rather than ones that match the context of the conversation. Understanding natural behaviors, biological processes that underlie them, and creating efficient algorithms to implement a convincing simulation via an ECA is challenging but critical to the success of future ECA-based self-management support [15,35].

### Strengths and Limitations

This mixed methods study is one of the first to explore users’ experiences of a sophisticated ECA in a real-world setting over a 6-month period and offers several novel findings and suggestions for future research. Although conducted within the context of a randomized controlled trial, our participants used the app in the context of their everyday lives, which is a strength of the research. The mixed methods approach provides robust evidence based on responses from a wide range of participants. However, people who were retired, highly educated, and engaged with the app were overrepresented in the interviewed sample of participants.

### Conclusions

The importance of the relational aspect of agents for health care is becoming an increasingly prominent theme in the literature. Our study adds to this literature by describing the long-term experiences of people using an ECA for diabetes self-management support and making recommendations for improvements and future research. These findings suggest that ECAs play a promising role in self-management support and education. However, accommodating user preferences and expectations of the role that an ECA may play in self-management and improving their natural communication are key to their success.

## Appendix

Multimedia Appendix 1 Responses to question Q1: How well do the words below describe Laura? (N=66 ).

Multimedia Appendix 2 Responses to question Q2: How did interacting with Laura make you feel? (N=66 ).

## Abbreviations

ECA: embodied conversational agent
HbA1c: glycated hemoglobin A1c
MDC: My Diabetes Coach
NDSS: National Diabetes Services Scheme
T2D: type 2 diabetes
