# Supplementary figures and images for: Acceptability of an Embodied Conversational Agent for Type 2 Diabetes Self-Management Education and Support via a Smartphone App: Mixed Methods Study
Source: JMIR Mhealth Uhealth. 2020 Jul 22;8(7):e17038. doi: 10.2196/17038 (PMC7407258; doi:10.2196/17038)

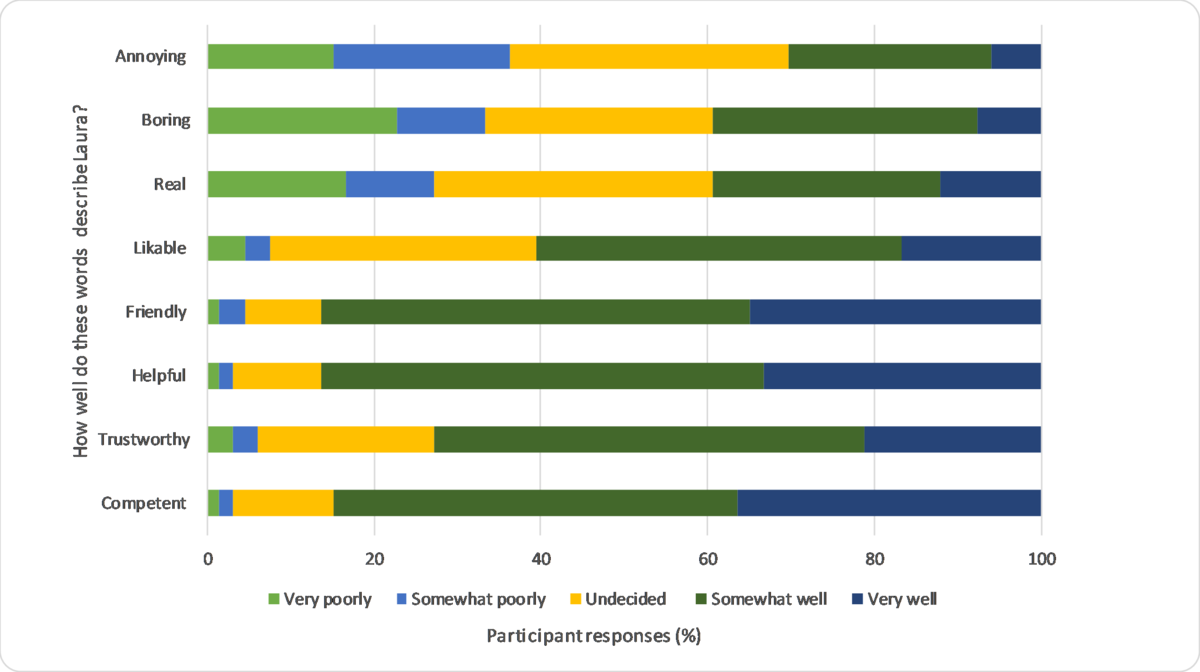

Supplement: Multimedia Appendix 1 [file mhealth_v8i7e17038_app1.png]

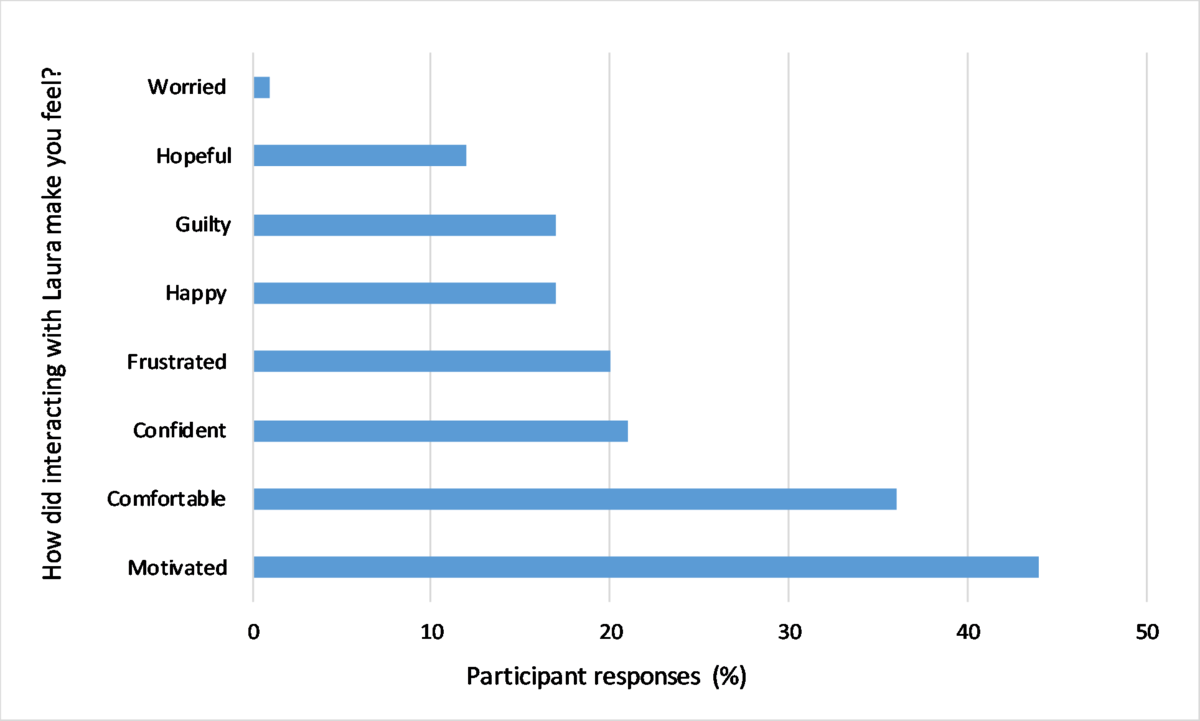

Supplement: Multimedia Appendix 2 [file mhealth_v8i7e17038_app2.png]
